# Biochemical and molecular insights into anthracnose resistance in chilli

**DOI:** 10.1371/journal.pone.0347774

**Published:** 2026-06-05

**Authors:** Manisha Mangal, Jameel Akhtar, Arpita Srivastava, Pardeep Kumar, Sushma Sagar, Theivanai Murugan, Purva Khandelwal, Shilpi Kumari, Vinod Kumar Sharma, Jai Chand Rana

**Affiliations:** 1 Division of Vegetable Science, ICAR-Indian Agricultural Research Institute, New Delhi, India; 2 Division of Plant Quarantine, ICAR-National Bureau of Plant Genetic Resources, New Delhi, India; 3 Division of Germplasm Evaluation, ICAR-National Bureau of Plant Genetic Resources, New Delhi; 4 The Alliance of Bioversity International and CIAT, New Delhi, India; Southern Federal University Academy of Biology and Biotechnology named after D I Ivanovsky: Uznyj federal’nyj universitet Akademia biologii i biotehnologii im D I Ivanovskogo, RUSSIAN FEDERATION

## Abstract

Hot pepper (*Capsicum annuum* L.) is a globally important vegetable crop valued for its nutritional, economic, and culinary significance, yet its productivity is severely threatened by the fruit-rot/anthracnose disease complex caused by *Colletotrichum* spp. (*C. capsici, C. gloeosporioides,* and *C. acutatum*). This study investigated the biochemical and molecular defense responses of two resistant genotypes, CH-281 (EC777200), resistant to *C. capsici*, and CH-319 (IC119695), resistant to *C. gloeosporioides*, in comparison with Kashi Anmol, which is susceptible to both pathogens.

Resistant genotypes showed smaller lesion diameters, higher activities of antioxidant enzymes (SOD, CAT, GPX, POX), and increased phenolic accumulation, indicating effective oxidative stress management and sustained defense. Gene expression analyses revealed early and strong induction of defense-related genes in resistant lines. CH-281 challenged with *C. capsici* exhibited rapid upregulation of PR1 (996-fold), WRKY33, WRKY40, CaMYB, ACS, AOS, and Defensin-like proteins, whereas Kashi Anmol displayed delayed and weaker responses with late surges in ACS and AOS. Similarly, against *C. gloeosporioides*, CH-319 showed early activation of PR1, WRKY33, CaMYB, and Defensin-like proteins, while the susceptible genotype again demonstrated delayed induction with stronger late peaks.

Overall, resistant genotypes mounted timely and robust biochemical and transcriptional defenses, in contrast to the susceptible genotype’s delayed responses. These findings highlight the molecular basis of anthracnose resistance and identify key defense genes and pathways that can be harnessed for breeding durable resistance in chilli.

## Introducton

Hot peppers (*Capsicum annuum* L.) are economically valuable and nutritionally rich vegetables widely cultivated across the globe. They are important components of many culinary traditions due to their characteristic tangy-pungent flavour, attributed to capsaicinoids [1]. In addition to their culinary appeal, they offer significant health benefits owing to their rich content of vitamins (A, C, B-complex, and E), minerals (such as calcium, phosphorus, and potassium), and bioactive compounds like carotenoids, flavonoids, and phenolic compounds [1–3]. Their high demand as both a spice and a vegetable has made chilli a vital global cash crop, contributing to food and nutritional security through enhanced farmer income and its richness in vitamins and antioxidants. [4].

Chilli cultivation is highly adaptable, flourishing in both temperate and tropical regions. It can be grown as either a perennial or annual crop depending on the climate, favouring well-drained loamy soils and temperatures ranging between 18–30°C [5].

Despite its agricultural and nutritional importance, chilli is highly vulnerable to biotic stresses, with anthracnose being one of the most devastating diseases. Chilli anthracnose, caused mainly by *Colletotrichum* spp., is a globally prevalent disease that severely impacts chilli cultivation. The pathogen is notorious for establishing latent infections that remain asymptomatic until fruit ripening, leading to extensive pre- and post-harvest losses. Globally, the disease has been reported from all major chilli-growing regions, with severe outbreaks in Southeast Asia [6–7], China and Korea [8], and also in Africa and Latin America (Mexico, Brazil) [9]. Across these regions, yield losses may reach 20–100%, depending on cultivar susceptibility, pathogen species, and environmental conditions [7]. Latent infections alone can reduce yields by up to 50% [6].

In India, anthracnose is prevalent across nearly all chilli-growing states, with severe incidences reported in Andhra Pradesh, Telangana, Karnataka, Tamil Nadu, Maharashtra, Madhya Pradesh, Rajasthan, Punjab, Haryana, and Assam [7,10,11]. Reported yield losses range from 10% to 54% [12–13], while marketable fruit losses are estimated between 10% and 80% due to both pre-harvest and post-harvest involvement of the pathogen [8]. The economic burden is considerable, with an estimated annual loss of 29.5% amounting to US$ 491.67 million reported for India alone [14]. The most economically impactful symptom is fruit rot, as even minor lesions can drastically reduce marketability and profitability [15]. The disease affects almost all aerial parts of the plant – including leaves, stems, and fruits at both green and mature stages – and can also infect seeds during storage [8,16,17]. *Colletotrichum capsici* and *C. gloeosporioides* are recognized as the major causal agents of chilli anthracnose in tropical Asia [15,18], Korea [19], and parts of the United States, including Louisiana and Mississippi [20–21].

In India, *Colletotrichum capsici*, *C. acutatum*, and *C. gloeosporioides* are commonly associated with anthracnose, with *C. capsici* and *C. gloeosporioides* causing the most severe crop damage [22–25]. These pathogens cause distinct symptoms, including dark, sunken necrotic lesions with concentric rings of acervuli, leaf spots, dieback, seedling blight, and damping-off. Moreover, the two major species infect at different developmental stages – *C. capsici* primarily during fruit ripening, while *C. gloeosporioides* infects both young and mature green fruits [8,26].

Due to the widespread occurrence and severity of anthracnose, understanding host-pathogen interactions is critical for managing the disease and ensuring stable chilli production. Plants have evolved intricate defense mechanisms to counter pathogen attacks. Upon recognizing pathogen-associated molecular patterns, plants activate a range of biochemical and molecular responses. These include the accumulation of phenolic compounds, flavonoids, lignin, and the induction of enzymes like phenylalanine ammonia-lyase (PAL), polyphenol oxidase (PPO), and antioxidant enzymes such as superoxide dismutase (SOD), catalase (CAT), and peroxidases (POX).

Phytohormones like auxins, gibberellins (GA), salicylic acid (SA), ethylene (ET), jasmonic acid (JA), and brassinosteroids play essential roles in regulating these defense responses. While SA-dependent pathways are typically involved in resistance against biotrophic and hemibiotrophic pathogens, JA and ET pathways are crucial in defense against necrotrophs [27]. In chilli, resistant genotypes challenged with *Colletotrichum* exhibit rapid induction of JA/ET-responsive genes (*LOX3, AOS, ACS2, PDF1.2*), selective activation of SA-associated genes (*PAL*), and upregulation of PR proteins, particularly PR2 (β-1,3-glucanase) and PR5 (thaumatin-like protein). Transcription factors such as WRKY33, MYB, NAC, EREBP, and bZIP further fine-tune these responses [1]. Similar patterns have been reported in other Solanaceae: in tomato, resistant lines inoculated with *Alternaria alternata* showed stronger induction of WRKY genes and PR proteins, along with elevated chitinase and β-1,3-glucanase activity [28]; in potato, resistant genotypes challenged with *A. alternata* displayed higher expression of *PR1, PR2, PR5, ChtA, PAL,* and enhanced antioxidant enzyme activities (SOD, POX, PPO) compared to susceptible ones [29]. These studies highlight that conserved defense components—hormone signaling, PR gene activation, antioxidant regulation, and transcriptional control—collectively determine resistant versus susceptible outcomes across Solanaceae crops.

With this background, the present study was designed to investigate the differential biochemical and molecular responses of resistant and susceptible chilli genotypes to anthracnose disease caused by *Colletotrichum capsici* and *C. gloeosporioides*. The specific objectives were to elucidate the host–pathogen interactions underlying resistance and susceptibility and to generate insights that can facilitate the development of effective disease management strategies, thereby support sustainable chilli production and contributing to food and nutritional security.

## Materials and methods

### Plant materials and pathogen cultures

The experiment involved evaluation of response of chilli germplasm against two species of *Colletotrichum viz*., *C. capsici* and *C. gloeosporioides* in three chilli genotypes – Kashi Anmol (susceptible to both the species), CH-281 (EC777200) resistant to *C. capsici* and CH-319 (IC119695) resistant to *C. gloeosporioides.* Kashi Anmol is a medium-duration, high-yielding variety with pendant green fruits (~7.0 cm), maturing in ~60 days and producing about 180 q ha^−1^, with moderately tall and spreading plants. CH 319 is an early, compact genotype flowering in ~35 days, bearing tiny green fruits (~2.2 cm) while CH 281 is a medium-duration type flowering in ~40 days, producing attractive pendant medium-sized fruits (~8.2 cm) on taller plants with narrower spread. The resistant and susceptible genotypes used in the present study were selected based on previous screening, in which 200 chilli accessions were evaluated against *C. capsici* and *C. gloeosporioides* causing anthracnose*.*

Pure cultures of *C. capsici* and *C. gloeosporioides* were obtained from the Division of Plant Quarantine, ICAR-NBPGR, New Delhi and maintained on PDA medium supplemented with antibiotic, streptomycin (100 mg/L) to prevent bacterial contamination at 4 °C for further use.

### Pathogenicity test

Chilli fruits were collected during the winter season (January – February, 2024) from field and sterilized using 1% sodium hypochlorite solution followed by rinsing with sterilized water and drying with sterilized filter paper. After drying, fruits were spot inoculated by placing 10 μl conidial suspension (3–4 conidia/ μl) of *C. capsici* in water gelatine (2.0%) using micropipette [30]. Conidial concentration was adjusted by haemocytometer and gelatin was used to avoid the over spreading of the conidial droplets. The inoculated fruits were incubated at 22 ± 1°C and observed daily for 2 weeks to record infection and symptom development. Re-isolation was made on potato dextrose agar for further identification of the pathogen to compare with original culture used for inoculation under microscope to prove Koch postulates. The pathogenicity was proved and the identification was confirmed before its use for experimental purpose.

### Screening procedure

Conidia of the fungus were collected from actively growing ten days old culture with the help of scalpel. The spore mass was dissolved in distilled water and homogenised to get uniform concentration of spore suspension. The concentration of spore suspension was adjusted to 1x10^5^ conidia per ml of water with the help of haemocytometer. A set of six matured green chilli fruits were taken in each genotype. Fruits were carefully detached from plants and washed with sterile distilled water (SDW) and then wiped with cotton wools soaked in ethanol to remove surface contaminants. Fruits were spot inoculated by placing 10 μl conidial suspension (1 × 10^3^ conidia ml^-1^) of *C. Capsici* / *C. gloeosporioides* as prescribed earlier [30]. The post-inoculation observations were carried out as per Montri et al [31]. Inoculated fruits were maintained for 2 weeks in plastic moisture boxes lined with moistened blotter paper under high humidity (95–98%), a temperature of 26°C, and a 12-hour light/dark cycle [32].

To examine the differential biochemical and molecular responses of chilli genotypes with specific resistance, CH-281 (resistant only to *C. capsici*) was challenged with *C. capsici*, while CH-319 (resistant only to *C. gloeosporioides*) was challenged with *C. gloeosporioides*. Since the susceptible genotype Kashi Anmol was found susceptible to both pathogens (in prevous study), it was used as a common susceptible check and inoculated with both the anthracnose species in respective sets. As anthracnose is not commonly prevalent under field conditions in Delhi, and in the absence of specialized protected cultivation facilities for artificial challenge inoculation at the whole-plant level, the experiments were conducted using detached chilli fruits. The sampling time points were selected based on the distinct disease progression patterns of *C. capsici* and *C. gloeosporioides* in the respective host genotypes, ensuring that critical stages of pathogen establishment, lesion development, and host responses were adequately captured for both pathosystems. For *C. capsici*, fruit samples from Kashi Anmol and CH-281 were collected at 0 hr (control), 24 hr, 48 hr, 72 hr, 144 hr, 288 hr, and 336 hr post-inoculation while for *C. gloeosporioides*, samples were taken from CH-319 and Kashi Anmol at 0 hr, 24 hr, 48 hr, 72 hr, 144 hr, 192 hr, and 240 hr. All samples were immediately frozen at −80°C for further analysis.

### Morphological parameter

Lesion diameter was measured on the fruit surface to track disease progression. Three replicates each with two fruits were used per genotype, and lesion sizes were recorded at each sampling interval for morphological assessment.

### Biochemical parameter studied for anthracnose

#### Preparation of enzyme extract.

Infected samples for biochemical and molecular studies comprised fruit sections 1 cm above and below the point of inoculation. To extract enzymes, 1 gm of infected fruit tissue was homogenized in 10 ml of extraction solution (0.1 M phosphate buffer (pH 7.0), 0.5 mM EDTA, and 1% polyvinylpyrrolidone (PVP)) using pre-chilled mortar and pestle on ice. The homogenate underwent a 30 minute, 12,000g centrifugation at 4°C. After that, the supernatant was gathered and put to use in enzymatic activity tests for guaiacol peroxidase (GPX), catalase (CAT), peroxidase (POX) and superoxide dismutase (SOD).

#### Catalase (CAT).

The method described by Aebi [33] was used to measure the catalase activity by observing the breakdown of hydrogen peroxide (H_2_O_2_) at 240 nm. Triple-distilled water was used to adjust the final volume of the modified reaction mixture to 3 mL. It contained 1.5 mL potassium phosphate buffer, 0.5 mL H_2_O_2_, and 50 μl of enzyme extract. After adding H_2_O_2_ to start the process, the absorbance at 240 nm decreased for a duration of one minute. Comparing the absorbance results with a standard curve of known H_2_O_2_ concentrations allowed for the quantification of the quantity of H_2_O_2_ that had decomposed. Reduction in H_2_O_2_ concentration (μmole/min/g FW) was used to express catalase activity.

#### Superoxide dismutase (SOD).

The SOD activity was measured by measuring the inhibition of nitroblue tetrazolium (NBT) reduction at 560 nm using the protocol outlined by Dhindsa et. al [34]. The reaction mixture (3 mL) consisted of 0.2 mL methionine, 0.1 mL NBT, 0.1 mL EDTA, 1.5 mL phosphate buffer, 0.1 mL sodium carbonate, 0.05–0.1 mL enzyme extract, and double-distilled water to make up the volume. The reaction was started by adding 0.1 mL of 60 μM riboflavin and was then illuminated for 15 minutes under two 15 W fluorescent lamps. To generate the maximum colour, a control consisting of the entire reaction mixture without the enzyme was used. The reaction was stopped by placing the tubes in the dark. The blank was a non-irradiated mixture. The absorbance at 560 nm was measured, and one unit of SOD activity was defined as the enzyme concentration that caused a 50% reduction in absorbance compared to the control.


$$\begin{array}{c} \text{Enzyme activity }(\text{units}/\text{g FW}/\text{min})\;=\;\frac{\text{Control }-\;(\text{Sample }-\text{ Blank})}{\text{2}} \end{array}$$

#### Guaiacol Peroxidase (GPX).

Following Everse et al [35] guaiacol peroxidase activity was determined by observing the tetra-guaiacol production at 470 nm. To make the reaction mixture with final volume 3 mL, the following ingredients were added: 0.5 mL guaiacol (16 mM), 1.0 mL phosphate buffer (50 mM, pH 6.1), 0.5 mL H_2_O_2_ (2 mM), 0.1 mL enzyme extract, and 0.9 mL water. The extinction coefficient for tetra-guaiacol (ε = 26.6 mM^-1^ cm^-1^) was used to compute the enzyme activity, which was then represented as micromoles of tetra-guaiacol produced per minute per gram of fresh weight.

#### Peroxidase activity (POX).

The POX activity was carried out utilizing the approach of Hammerschmidt et al [36]. Chilli fruit (500 mg) was pulverized for this experiment at 4 °C in a cooled mortar and pestle with 3 mL of 100 mM sodium phosphate buffer (pH 7.0). The extracted solutions were centrifuged at 16,000 rpm for 15 minutes. The enzyme extract was made from the aqueous solution. The reaction mixture of 2.5 mL was created by adding 1.5 mL of 50 mM pyrogallol, 0.5 mL of 1% hydrogen peroxide, and 0.5 mL of crude enzyme extract. For a minute, spectrophotometric measurements of the variations in optical density were made at 470 nm. The expression of the peroxidase activity was μmol min − 1 mg − 1 protein, with an extinction coefficient of 26.6 mM − 1 cm − 1. For each treatment, the experiment was run three times in duplicate.

#### Total phenol content determination.

The method of Swain and Hillis with modification by Bhatia et al [37,38] was used to quantify the total phenol content. 200 mg of plant tissue were crushed in 2 mL of 80% ethanol for each sample, and the mixture was centrifuged at 5,000 rpm for 20 minutes at 4°C. 100 μl of the enzyme extract, 2.9 mL of distilled water, and 0.5 mL of the Folin-Ciocalteu reagent that had been refrigerated beforehand were combined in a test tube, and the combination was left in the dark for five to ten minutes. After adding 2 milliliters of 20% Na_2_CO_3_, the reaction mixture was allowed to sit for 60 minutes. Using a blank for the reagent, the absorbance was measured at 750 nm. The standard curve was prepared using gallic acid as the working standard solution (100 µg mL ⁻¹) at concentrations ranging from 10 to 100 µg, and the total phenolic content was calculated using the following formula:


$$\begin{array}{c} \text{Total}\;\text{phenolics}\;(\text{mg}/\text{100}\;\text{g})=\frac{\text{A}\times \text{Total extract volume}\;(\text{ml})\times \text{100}}{\text{Aliquot}\;(\text{ml})\times \;slope\;of\;standard\;curve\;x\;\text{weight}\;\text{of}\;\text{sample}\;(\text{g})\times \text{1000}} \end{array}$$

### Gene expression analysis

#### RNA isolation and cDNA synthesis.

Infected fruit samples collected at various time points were promptly stored at −80°C until further processing. RNA was extracted from 100 mg of infected fruit tissue using the Tri-Xtract reagent (G Biosciences, USA), adhering to the manufacturer’s protocol. RNA quality and concentration were assessed via nano-spectrophotometry and denaturing agarose gel electrophoresis to ensure the integrity of the samples. Expression data were analyzed using three biological and two technical replicates, ensuring the robustness of the results. The Verso cDNA Synthesis Kit (Thermo Fisher Scientific, Inc.) was used to reverse transcribe 1 μg of high-quality RNA into complementary DNA (cDNA). Following this, 1 μl of the tenfold diluted cDNA was utilized as a template for the quantitative real-time PCR (qRT-PCR) experiments. A final volume of 10 μl was used for each qRT-PCR reaction, which contained 1 μl of the cDNA template, 3 μl of nuclease-free water, 0.5 μl of each of the forward and reverse primers (100 nM), and 5 μl of 2 × SYBR Green Master Mix (Applied Biosystems, CA, USA). The following parameters were applied to reactions conducted on Roche Life Sciences’ LightCycler® 96 Real-Time PCR System: initial denaturation at 95°C for 120 seconds, followed by 40 cycles of denaturation at 95°C for 30 seconds, annealing at 55°- 60°C for 60 seconds (depending upon the primer), and extension at 72°C for 30 seconds.

For the normalization of gene expression levels, ubiquitin (UBQ) was used as the internal reference gene. The ΔCt method was employed to normalize the expression values of target genes relative to UBQ. To determine fold changes in gene expression, the ΔΔCt method was applied, using the ΔCt values from the not inoculated fruit of each genotype as a callibrator. Although some of the information regarding primers sequences used for quantitative PCR (qPCR) to investigate gene expression profiles associated with *Colletotrichum* infection in chilli genotypes (Tables 1) were taken from the published data by [1] however, details regarding CDS Length, mRNA length, Primer binding position in the CDS, amplicon length, Primer efficiency, limit of detection, primer melting and annealing temp were calculated using following methodology. CDS length and mRNA length was obtained from the NCBI (https://www.ncbi.nlm.nih.gov/nuccore/) by searching against the accession number, primer binding position in the CDS were obtained from the online available tool primer map (http://www.genecorner.urgent.be), Amplicon Length was calculated using the following formula:


$$\begin{array}{c} 𝐀𝐦𝐩𝐥𝐢𝐜𝐨𝐧\;𝐋𝐞𝐧𝐠𝐭𝐡=𝐄𝐧𝐝\;𝐩𝐨𝐬𝐢𝐭𝐢𝐨𝐧\;𝐨𝐟\;𝐑𝐞𝐯𝐞𝐫𝐬𝐞\;𝐩𝐫𝐢𝐦𝐞𝐫-𝐒𝐭𝐚𝐫𝐭\;𝐩𝐨𝐬𝐢𝐭𝐢𝐨𝐧\;𝐨𝐟\;𝐅𝐨𝐫𝐰𝐚𝐫𝐝\;𝐩𝐫𝐢𝐦𝐞𝐫+1 \end{array}$$

**Table 1:** Sequences of gene specific primer pairs used for quantitative real time polymerase chain reaction experiments.

S. No	Gene	Accession no.	Complete name in Gene bank	Forward primer	Tm (FP)	Reverse primer	Tm (RP)	Primer binding position in the CDS/mRNA	CDS Length	Gene length	Amplicon length	Primer efficiency (%)	LOD	Annealing temp
**1.**	Allene oxide synthase (AOS)	DQ832720	*Allene oxide synthase* mRNA	5′- CCGTTCTGTCATTTCATCCA-3′	55.2	5′- TTCAATGCGGAGAGATTCGT-3′	56.2	F-150–169 R-230–249	536 bp	–	100	103.83	10ng/μl	55.2
2.	PR-1	AY560589	*Capsicum annuum* basic PR-1 protein precursor, gene	5′- TGGTGTCGGCCCTATGACA-3′	60.6	5′- GGCCACCAGAGTGTTGCAT-3′	59.7	F- 2765–2783 R-2847–2865	3428 bp	–	101	106.91	10ng/μl	59.7
3.	WRKY33	AY789641	*Capsicum annuum* WRKY family transcription factor mRNA, complete cds GenBank: AY789641.1	5′- GTCCTACCGGTGGCAATAGC-3′	59.8	5′- TGCTTTGAAGCTTGGATCTTTG-3′	58.4	F-986–1005 R-1089–1110 (of mRNA)	1086 bp	1646 bp mRNA	125 bp	100.92	10ng/μl	58.4
4.	CaMYB	EU560899	*Capsicum annuum* clone DEG202 MYB transcription factor mRNA, complete cds	5′- CGCAAGCGAGGAGTTCCAT-3′	59.6	5′- TGTGTCGGTGTGCGAGTCTT-3′	60.7	F-362–380 R-467 (of mRNA)	846 bp	1020 bp mRNA	125 bp	109.3416	10ng/μl	59.6
5.	WRKY 40	NM_001325081.1	*Capsicum annuum* probable WRKY transcription factor 40 (LOC107876015), mRNA	5′- TCAATCCTTCAGGACCAACCA-3′	61.4	5′- CCACCGGTAGGACTAGCACTCT-3	64.5	F-899–919 R-977–998 9 of mRNA)	777 bp	1442 bp mRNA	100	104.7403	10ng/μl	61.4
6.	ACC synthase 2 (ACS2)	XM_016682648	*Capsicum annuum* *1-aminocyclopropane-1-carboxylate synthase 2* (LOC107839239), mRNA	5′- TTCCAAATTACCGCAAAAGC-3′	54.2	5′- GTGGATTTGATGGGTTGGTC-3′	56.7	F-841–860 R-921–940 9 of mRNA)	1460 bp	1961 bp mRNA	100 bp	99.52623	10ng/μl	54.2
7.	CaNAC	JX402928	*Capsicum annuum* NAC domain class transcription factor mRNA	5′- CCTCCTAACGGTGACAGATATGC-3′	61.5	5′- CCCTCGACTTGCGCTTCAT-3′	59.6	F 659–681 R-765–783 (of mRNA)	1230 bp	1490 bp mRNA	125 bp	100.9233	10ng/μl	59.6
8.	bZIP10	AY775332	*Capsicum annuum* bZIP transcription factor protein (bZIP1) mRNA	5′-CCGTGCTCTTGTTGGAATCC-3′	58.8	5′- TCTGGAATTGACCAGGTTTGG-3′	60.5	F-620–639 R-699–719 9 of mRNA)	861 bp	1122 bp mRNA	100 bp	108.8476	10ng/μl	58.8
9.	Defensin Like protein	XM_047403439.1	flower-specific defensin-like, LOC124891865	5′- TGCTGTAAAGTGCCCACAACA-3′	61.4	5′- TGTCGAAACAATGACCATCTTCA-3′	59.4	F-176–196 R-253–275 9 of mRNA)	276 bp	mRNA 564 bp	100	99.52623	10ng/μl	59.4
10.	Ubiquitin 3 (UBI3)	AY486137	*Capsicum annuum* ubiquitin-conjugating protein mRNA, complete cds	5′- TCAAGCCTCCAAAGGTTGCT-3′	58.7	5′- GGACTCCACTGCTCCTTGAGA-3′	63.6	F117 to 136 R-196–216 9of mRNA)	360 bp	578 bp mRNA	100	105.3525	10ng/μl	58.7

Primer melting and annealing temperatures were calculated using Tm calculator tool at (https://www.thermofisher.com) while Primer efficiency and limit of detection was calculated as mentioned below.

#### Primer efficiency.

Primer efficiency was calculated using a 10-fold serial dilution of the template DNA, ranging from 1000ng/ μl template to 1ng/μl. qPCR reactions were set up for each dilution, and Ct values were recorded. A standard curve was plotted with Ct values on the y-axis and the log10 of template concentrations on the x-axis. The slope of the curve was determined using linear regression, and efficiency was calculated using the formula:


$$\begin{array}{c} \text{E}=({\text{1}\text{0}}^{-\text{1}/\text{slope}}-\text{1})\times \text{100} \end{array}$$

The efficiency values were interpreted to confirm optimal primer performance (90–110%).

#### Limit of detection.

The limit of detection (LOD) of a primer was determined using a 10-fold serial dilution of the template DNA, ranging from 1000ng/μl template to 1 ng/μl. The qPCR reactions were performed, and Ct values were recorded for each dilution. A Ct value of 35 was set as the detection threshold. The dilution factor at which the Ct value was below 35 was identified as the LOD. The corresponding DNA concentration (ng/μl) at this dilution represented the primer’s limit of detection.

### Statistical analysis

The experiment was conducted in a completely randomized block design, with each treatment replicated thrice. Analysis of variance (ANOVA) was performed using OPSTAT statistical software (http://14.139.232.166/opstat/).

## Results

### Morphological disease progression

**Fig 1:**
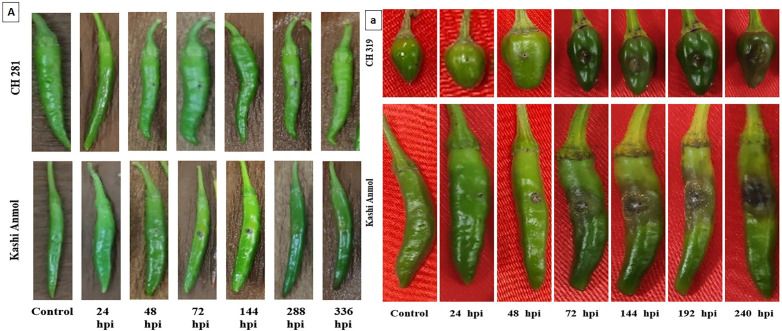
Phenotypic observations on the fruits of the susceptible and the resistant genotypes of Capsicum at different hours post-inoculation. **A**. Resistant genotype CH 281 and susceptible genotype Kashi Anmol infected with *Collectrotrichum capsici,* **a.** Resistant genotype CH 319 and susceptible genotype Kashi Anmol infected with *Collectrotrichum gloeosporioides.*

**Fig 2:**
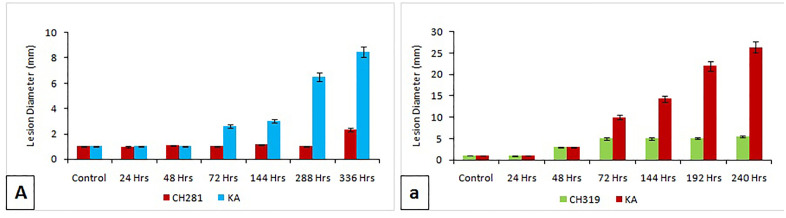
Morphological disease progression following infection in the test genotypes. **A.** *Collectrotrichum capsici* and **a.** *Collectrotrichum gloeosporioides.*

Lesion diameter differed significantly between the resistant genotype CH-281 and the susceptible genotype Kashi Anmol following inoculation with *C. capsici*. The resistant genotype CH-281 consistently displayed smaller lesion diameters with minimal variation. Conversely, the susceptible genotype Kashi Anmol exhibited a progressive increase in lesion diameter, with a marked expansion observed at 288 hpi, however there was peak at 336 hpi (p < 0.05). Infection with *C. capsici* at 14 DPI produced lesions measuring 8.5 mm in Kashi Anmol, while the resistant genotype CH-281 showed minimal lesion development, with a diameter of only 2 mm. Similarly, significant variation in lesion diameter was also observed between the resistant genotype CH-319 and the susceptible genotype Kashi Anmol following inoculation with *C. gloeosporioides*. In Kashi Anmol, infection with *C. gloeosporioides* resulted in a lesion diameter of 26.5 mm at 10 DPI, whereas the resistant genotype CH-319 exhibited a significantly smaller lesion diameter of 5.5 mm (Figs 1 and 2).

### Biochemical activities

**Fig 3:**
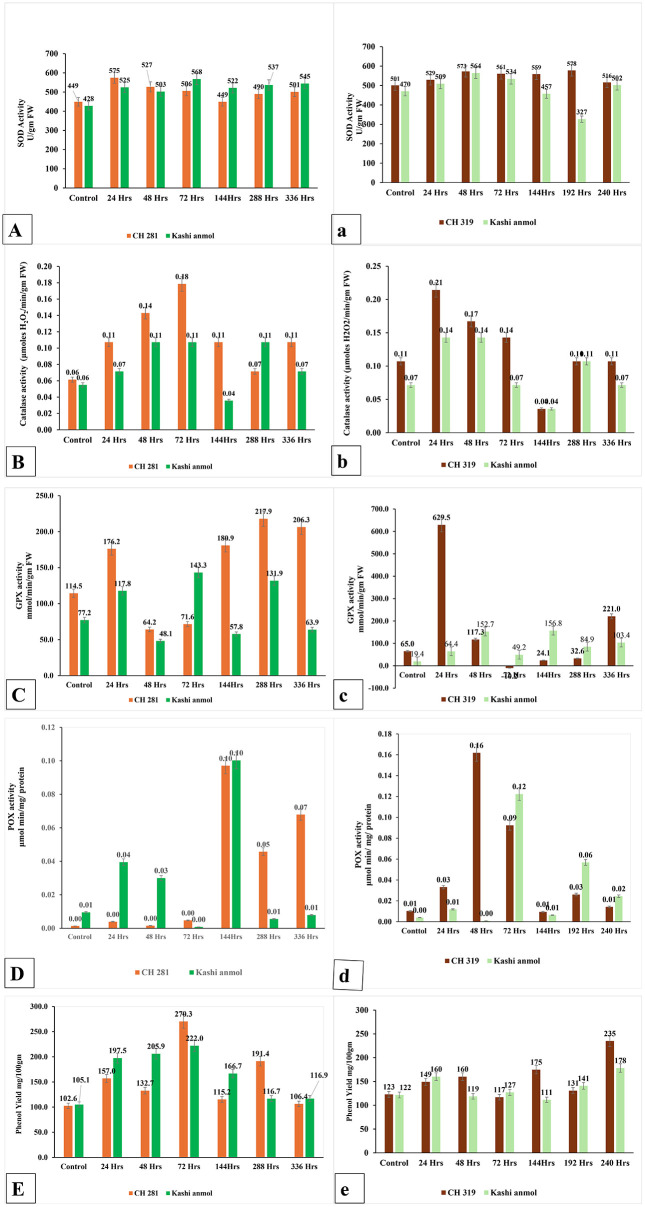
Activity of ROS scavenging enzymes in resistant and susceptible genotype at different time intervals post inoculation with *Collectrotrichum capsici* (A-E) and *Collectrotrichum gloeosporioides* (a-e)(X- axis- different time intervals post inoculation, Y- axis- enzyme activity).

Biochemical profiling of chilli fruits inoculated with *Colletotrichum capsici* and *C. gloeosporioides* revealed pronounced temporal and genotypic differences in the activity of antioxidant enzymes and phenolic accumulation (Fig 3).

#### Superoxide dismutase (SOD).

Following *C. capsici* infection, SOD activity increased sharply in both genotypes peaking at 24 hpi in CH-281 (575 U/g FW) and Kashi Anmol (525 U/g FW). Thereafter, activity declined progressively, with CH-281 maintaining higher levels up to 48 hpi, while Kashi Anmol exhibited relatively higher activity from 72 hpi onward (568 U/g FW at 72 hpi). In contrast, *C. gloeosporioides* inoculation triggered a gradual and sustained increase in SOD activity in CH-319, peaking at 192 hpi (578 U/g FW). Kashi Anmol exhibited lower activity overall, although a transient peak was observed at 48 hpi (564 U/g FW). These results suggest that resistant genotypes mount an earlier and more consistent SOD response particularly against *C. gloeosporioides*, whereas the susceptible genotype displays delayed or transient induction.

#### Catalase (CAT).

CAT activity exhibited similar trends in both genotypes after *C. capsici* inoculation, increasing steadily until 72 hpi. However, the magnitude of induction was greater in CH-281, with a peak of 0.18 μmoles H_2_O_2_/min/g FW at 72 hpi, compared to 0.11 μmoles in Kashi Anmol. Although activity declined thereafter, CH-281 generally maintained higher levels, except at 288 hpi. Under *C. gloeosporioides* challenge, CH-319 displayed a rapid and strong induction of CAT at 24 hpi (0.21 μmoles H_2_O_2_/min/g FW), followed by a gradual decline, whereas Kashi Anmol showed consistently lower activity, with only modest fluctuations (maximum 0.14 μmoles H_2_O_2_/min/g FW at 24 & 48 hpi). The enhanced CAT activity in resistant genotypes indicates a more efficient ROS detoxification mechanism.

#### Guaiacol peroxidase (GPX).

In response to *C. capsici*, GPX activity did not follow a distinct trend but remained consistently higher in CH-281 compared with Kashi Anmol at most time points, except at 72 hpi when Kashi Anmol showed greater activity (143.3 mmol/min/g FW). The maximum level in CH-281 was observed at 288 hpi (217.9 mmol/min/g FW). Following *C. gloeosporioides* inoculation, CH-319 exhibited a sharp induction at 24 hpi (629.5 mmol/min/g FW), which declined rapidly thereafter. Kashi Anmol showed only a modest rise, peaking at 48 hpi (152.7 mmol/min/g FW), before decreasing. These results suggest that rapid and strong GPX activation may be a key component of resistance to *C. gloeosporioides*.

#### Peroxidase (POX).

The POX activity in the resistant genotype (CH-281) showed a delayed response against *C. capsici* and displayed stronger and higher response than the susceptible Kashi Anmol only after 144 hpi when it showed maximum activity. In contrast, Kashi Anmol displayed higher activity during early time points (0–48hpi). Under *C. gloeosporioides* infection, the trend was reversed: CH-319 exhibited strong activity at early stages, with a sharp spike at 48 hpi, while Kashi Anmol showed a delayed response, peaking at 72 hpi. In both genotypes, POX activity declined after 144 hpi, though CH-319 maintained relatively higher levels.

#### Phenolics.

Phenolic content increased significantly in both genotypes after *C. capsici* infection upto 72 hpi however no specific trend was observed between resistant and susceptible genotypes where in Kashi Anmol showed strong induction at earlier time points upto 48hpi while CH-281 showed consistently higher accumulation thereafter. The highest content was recorded at 72 hpi (270.3 mg/100g in CH-281 vs. 222.0 mg/100g in Kashi Anmol). Similarly, under *C. gloeosporioides* challenge, CH-319 accumulated greater amounts of phenolics than the susceptle genotype with peak values at 240hpi (235 mg/100g), compared to Kashi Anmol (179 mg/100g). The elevated phenolic accumulation in resistant genotypes underscores their contribution to structural defense and antimicrobial activity.

Overall, resistant genotypes (CH-281 and CH-319) exhibited stronger and more sustained activation of antioxidant enzymes and greater phenolic accumulation than the susceptible genotype Kashi Anmol, highlighting their critical role in mitigating oxidative stress and conferring resistance against *Colletotrichum* infections.

### Gene expression analysis

**Fig 4:**
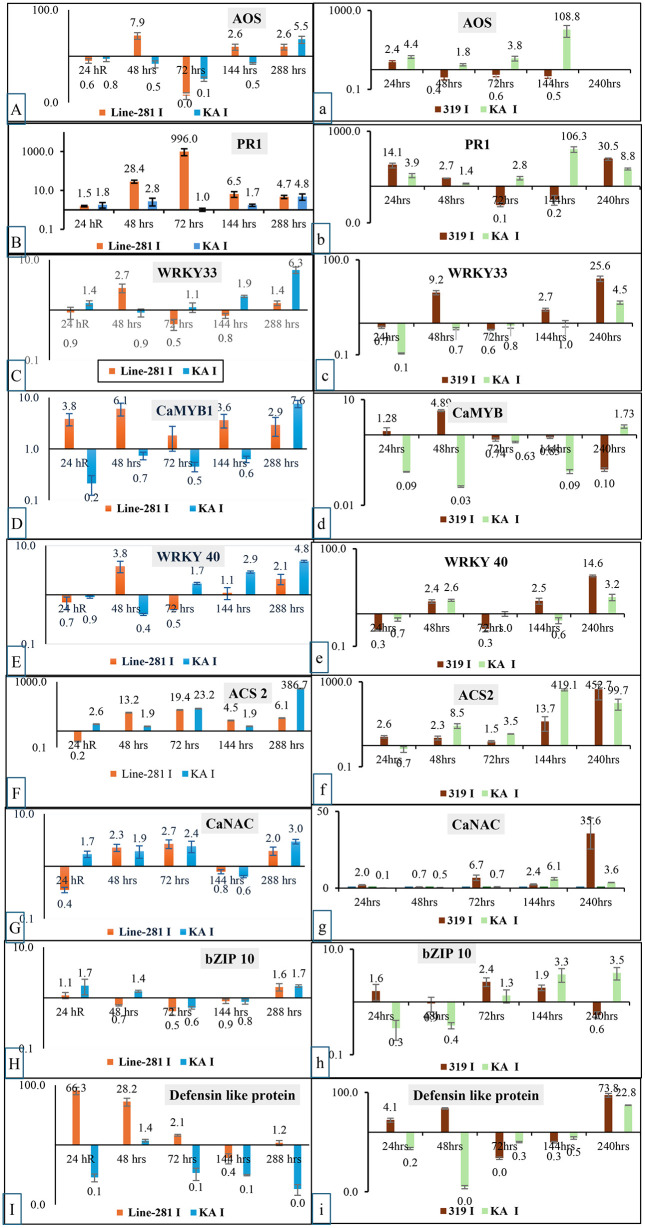
Relative fold change in the expression of different defense genes post inoculation with *Collectrotrichum capsici* (A-I) and *Collectrotrichum gloeosporioides* (a-i). (X- axis- different time intervals post inoculation, Y- axis- relative fold change in the expression of genes).

The expression patterns of defense-related genes were analyzed in chili fruit samples from two resistant genotypes—CH281 (resistant to *Colletotrichum capsici*) and CH319 (resistant to *C. gloeosporioides*)—as well as the susceptible genotype Kashi Anmol, following inoculation with the respective pathogens at different post-inoculation time points. The findings revealed distinct temporal and genotypic differences between resistant and susceptible chilli genotypes in response to *C. capsici* and *C. gloeosporioides* infection, particularly for key defense-associated genes including *Allene oxide synthase* (*AOS*), *PR-1*, *WRKY33*, *CaMYB 1*, *WRKY 40, ACC synthase 2*(*ACS*), *CaNAC*, *bZIP10* and *Defensin Like protein*, (Fig 4).

#### Gene expression profile against *C. capsici.*

The resistant genotype CH-281 exhibited early and strong induction of several defense-related genes compared with the susceptible genotype Kashi Anmol. *AOS* expression increased markedly at 48 hpi (7.9-fold) in CH-281, whereas Kashi Anmol showed only marginal induction. *PR1* was strongly upregulated in CH-281, reaching a dramatic peak at 72 hpi (996-fold), while Kashi Anmol maintained very low expression levels throughout. *WRKY33* showed slight upregulation in susceptible genotype Kashi Anmol (1.4-fold at 24 hpi), however the expression remained negligible in Kashi Anmol until a late rise at 288 hpi (6.3-fold), on the other hand CH281 showed strong induction of WRKY33 at 48hpi. *CaMYB* was consistently induced at higher levels in CH-281, peaking at 48 hpi (6.1-fold), while Kashi Anmol showed only weak induction at later stages (288hpi). Similarly, *WRKY40* expression was markedly higher in CH-281, especially at 48 hpi (3.8-fold), compared with suppression in Kashi Anmol where the expression was induced at later stages. *ACS* expression also followed this trend, with sustained induction in CH-281 (up to 19.4-fold at 72 hpi), whereas Kashi Anmol showed a sharp late induction at 288 hpi (385.7-fold), suggesting a delayed response. *CaNAC* was moderately upregulated in CH-281 (up to 2.7-fold at 72 hpi), while Kashi Anmol showed only slight increases. *bZIP10* expression remained low in both genotypes, Kashi Anmol had higher induction at earlier stages as compared to CH281. Notably, *Defensin-like protein* transcripts were strongly induced in CH-281 at early time points (66.3-fold at 24 hpi and 28.2-fold at 48 hpi), whereas Kashi Anmol exhibited consistently low expression. In general, the resistant genotype showed an early and strong induction of defense genes and transcription factors as compared to the susceptible one.

#### Gene expression profile against *C. gloeosporioides.*

In the resistant genotype CH-319, *AOS* expression was only modestly induced, while Kashi Anmol showed a consistently higher expression as compared to Kashi Anmol and displayed a very strong late peak at 240 hpi (108.8-fold), indicating delayed defense activation. *PR1* transcripts were more abundant in CH-319 at early time points (14.1-fold at 24 hpi), but Kashi Anmol showed a higher expression surge at 144 hpi (106.3-fold), again suggesting a slower but stronger late response. *WRKY33* was highly induced in CH-319 at 48 hpi (9.2-fold) and maintained a higher induction as compared to Kashi Anmol at all time points except at 72 hpi, whereas Kashi Anmol showed induction later at 240 hpi (4.5-fold) but it was still lower than Ch319. *CaMYB* exhibited early induction in CH-319 (4.9-fold at 48 hpi), while expression in Kashi Anmol remained suppressed at early stages but rose slightly by 240 hpi (1.73-fold). In contrast, *WRKY40* was strongly induced in CH-319 at late stages (14.6-fold at 240 hpi), whereas Kashi Anmol showed only minor increases. *ACC* transcripts were highly abundant in both genotypes, but the magnitude was greater in Kashi Anmol upto 144 hpi (419-fold at 144hpi), while CH319 peaked at 240 hpi (452.7-fold). *CaNAC* was strongly induced in CH-319 at 240 hpi (35.6-fold), whereas expression in Kashi Anmol remained comparatively lower. *bZIP10* showed higher expression in CH-319 upto 72hpi, while Kashi Anmol showed gradual upregulation thereafter, peaking at 240 hpi (3.5-fold). Finally, *Defensin-like protein* was strongly expressed at early time points in CH-319 at 48 hpi (15.8-fold), whereas Kashi Anmol showed higher late expression at 240 hpi (73.8-fold).

Overall, resistant genotypes (CH-281 and CH-319) were characterized by early and robust induction of key defense genes such as *PR1, WRKY40, WRKY33, CaMYB,* and *Defensin-like protein*, which are associated with pathogen recognition, transcriptional regulation, and antimicrobial activity. In contrast, the susceptible genotype Kashi Anmol generally exhibited delayed or suppressed responses, with late surges in *ACS* and *AOS*, reflecting an ineffective or slow defense strategy.

## Discussion

Devastative nature of the anthracnose/ fruit rot disease complex, and decade long survival of the fungus in seeds [17]. have been bottle neck in chilli cultivation and invited attention of the chilli researchers.

Breeding for resistance to anthracnose/ fruit rot disease complex has emerged as a critical objective in Capsicum improvement programs due to the recurring and widespread losses inflicted by *Colletotrichum* spp., which adversely affect both fruit quality and yield. Achieving durable resistance, however, necessitates an in-depth understanding of the morphological, biochemical, and molecular mechanisms that govern host-pathogen interactions.

Genetic resources possessing strong disease resistance serve as a crucial foundation for developing elite varieties [19]. Several studies have evaluated anthracnose resistance in diverse pepper germplasm to identify resistant sources [20–22]. In this study, susceptible genotypes exhibited rapid proliferation of the pathogen and extensive lesion development during the advanced stages of infection, in stark contrast to the restricted lesion formation in resistant genotypes. This contrast underscores the differential activation of defense responses among genotypes. Notably, variations in host responses were not limited to genotype differences alone but were also pathogen-specific, with distinct responses observed upon inoculation with *C. capsici* and *C. gloeosporioides*. These findings align with earlier reports emphasizing the race-specific nature of host-pathogen dynamics in *Capsicum* [8,39] and underscore the importance of considering pathogen diversity during resistance evaluation and breeding.

Plant immune responses are multi-faceted and initiate upon recognition of pathogen-associated molecular patterns (PAMPs) through pattern recognition receptors (PRRs), leading to PAMP-triggered immunity (PTI) [39,40]. PTI is followed by a more robust, intracellular response known as effector-triggered immunity (ETI), which is typically associated with hypersensitive response (HR) and localized cell death to confine the pathogen [41,42]. The production of reactive oxygen species (ROS) is a hallmark of these early defense responses, facilitating both antimicrobial action and signaling for downstream defense pathways [43]. At moderate levels, ROS contribute positively by reinforcing cell walls and inhibiting pathogen proliferation. However, their highly cytotoxic nature can disrupt normal cellular metabolism when present in excess. To mitigate severe oxidative damage to lipids, nucleic acids, and proteins caused by excessive ROS, plants have evolved a sophisticated antioxidant defense network [44,45]. This system includes enzymatic antioxidants such as peroxidase, superoxide dismutase, and catalase, along with enzymes of the ascorbate–glutathione cycle, including ascorbate peroxidase (APX), glutathione peroxidase (GPX), and others, which collectively facilitate ROS scavenging [46]. During stresses, SOD catalyses the removal of •O_2_^−^ by dismutating it into O_2_ and H_2_O_2_, CAT converts the H_2_O_2_ into water and molecular oxygen (O_2_) and POX works in the extra-cellular space for scavenging H_2_O_2_. Plant GPX catalyses the reduction of H_2_O_2_ and HO_2_ to water and lipid alcohols, respectively, using thioredoxin as an electron donor [47].

In the current study, SOD activity was significantly upregulated at 24–48 hours post-inoculation (hpi) with *C. capsici* in the resistant genotype CH-281, followed by a delayed response in the susceptible line Kashi Anmol. A similar trend was observed in GPX activity in CH-319 following *C. gloeosporioides* inoculation. SOD activity remained consistently high in resistant genotypes throughout *C. gloeosporioides* infection, while CAT activity was significantly elevated at all time points in resistant genotypes challenged with either pathogen. CAT activity exhibited different patterns in different plant species or genotypes under stress. In a study of sugarcane, the increase in CAT activity in resistant plants infected with a pathogen was detected, whereas the susceptible genotype did not alter the expression levels during the analysis [48]. Furthermore, GPX facilitates lignin formation by catalyzing the cross-linking of phenolics, which contributes to physical barrier formation—a crucial factor in combating hemibiotrophic pathogens like *Colletotrichum*.

In our study POX activity showed not only genotype dependent but also pathogen specific response as the response was contrasting against the two *Colletotrichum* species used in the present study. Earlier studies in *Capsicum* spp., have reported that elevated antioxidant enzyme activities were correlated with enhanced resistance [49] therefore our results are in line with earlier results.

Phenolic compounds play a pivotal role in plant defense. As lignin precursors, they strengthen cell walls at infection sites, thereby limiting pathogen ingress. In addition, phenolics function as signaling molecules that activate defense-related genes and contribute to systemic acquired resistance (SAR) [50]. In the present study, both genotypes exhibited an increase in phenolic content following inoculation, with a more pronounced response in resistant lines. This trend aligns with earlier reports in chilli challenged with *C. truncatum* [45,50]. Prasath and Ponnuswami [51] further demonstrated that resistant and moderately resistant genotypes of chilli infected with *C. capsici* accumulated significantly higher levels of ortho-dihydroxy phenols compared to susceptible ones.

At the molecular level, plant defense is regulated through hormone-mediated transcriptional reprogramming involving salicylic acid (SA), jasmonic acid (JA), and ethylene (ET). These signaling molecules activate a cascade of defense-related genes, including pathogenesis-related (PR) proteins and transcription factors. The current study revealed distinct gene expression profiles in resistant and susceptible genotypes depending on the infecting *Colletotrichum* species. Upon *C. capsici* inoculation, the resistant genotype CH-218 exhibited consistent upregulation of *Ca MYB*, Defensin like protein, and *PR3* genes, while *CaNAC* showed a similar pattern during *C. gloeosporioides* infection wherein genes such as *CaNAC,* and *ACS* were initially downregulated (24–48 hpi) but later upregulated in resistant lines, indicating a delayed but effective activation of defense pathways.

The expression of transcription factors and PR proteins was notably pathogen-specific. For example, *WRKY40* was strongly upregulated at 24–48 hpi in resistant genotypes challenged with *C. capsici*, but was activated later in susceptible lines. In contrast, *C. gloeosporioides* infection led to the significant induction of this gene in resistant line at most of the time points after 24hpi, highlighting differential defense gene activation. Similarly, b*ZIP 10*, Ca*MYB*, and *Defensin-like protein* exhibited enhanced expression in resistant lines following *C. gloeosporioides* infection. The *Defensin Like protein* also showed pronounced upregulation at early time points (24–48 hpi) in resistant genotypes, whereas these genes were downregulated in susceptible lines across the same time course.

These results echo earlier findings that *Colletotrichum* species can exploit diverse host signaling networks [1], but further demonstrate that both genotype and pathogen species exert substantial influence over the host response. This genotype × pathogen interaction is crucial to understanding the complex molecular dialogue governing resistance. Taken together, our findings highlight that resistance to anthracnose in chilli is not governed by a single defense pathway but rather by a dynamic interplay of antioxidative mechanisms, phenolic metabolism, and hormone-regulated gene expression, all of which operate in a genotype- and pathogen-specific manner. While earlier studies have emphasized the importance of antioxidant enzymes and phenolic compounds in conferring resistance, recent advances suggest that the durability of such resistance is often undermined by the high genetic variability and adaptive potential of *Colletotrichum* spp. [52]. This underscores a critical challenge: breeding efforts relying solely on single defense components may be insufficient, and integrating multi-omics approaches, including transcriptomics and metabolomics, is increasingly essential to dissect the complex host–pathogen interactions [53]. Furthermore, the strong genotype × pathogen interactions observed in this study reinforce the need for resistance screening against diverse races and species of *Colletotrichum* to ensure the development of broad-spectrum and durable resistance in Capsicum.

### Conclusion and implications

This study establishes that resistance to anthracnose in *Capsicum* is governed by a coordinated network of biochemical and molecular responses rather than by a single defense mechanism. Three critical insights emerge from our findings:

1. **Pathogen-specific and genotype-dependent responses:** The contrasting reactions of resistant and susceptible genotypes, and their differential responses to *C. capsici* and *C. gloeosporioides*, reaffirm the race-specific nature of host–pathogen interactions and highlight the necessity of evaluating resistance against diverse *Colletotrichum* populations.
2. **Early activation of defense markers in resistant genotypes:** Enhanced and timely induction of antioxidant enzymes (SOD, CAT, POX, GPX), phenolic accumulation, and key transcription factors underscores their central role in restricting pathogen progression and provides functional markers for resistance screening.
3. **Breeding and translational implications:** The resistant genotypes identified here represent valuable genetic resources that can be leveraged in gene pyramiding and molecular breeding to develop cultivars with broad-spectrum and durable anthracnose resistance, thereby strengthening the sustainability of chilli production.

By integrating pathogen diversity into screening and utilizing molecular tools to harness early defense markers, breeding programs can move beyond descriptive resistance evaluations toward the targeted development of resilient *Capsicum* cultivars
